# Gaze-Based Detection of Thoughts across Naturalistic Tasks Using a PSO-Optimized Random Forest Algorithm

**DOI:** 10.3390/bioengineering11080760

**Published:** 2024-07-27

**Authors:** Tarannum Rahnuma, Sairamya Nanjappan Jothiraj, Vishal Kuvar, Myrthe Faber, Robert T. Knight, Julia W. Y. Kam

**Affiliations:** 1 Department of Psychology, University of Calgary, Calgary, AB T2N 1N4, Canada; 2 Hotchkiss Brain Institute, University of Calgary, Calgary, AB T2N 4N1, Canada; 3 Department of Educational Psychology, University of Minnesota Twin Cities, Minneapolis, MN 55455, USA; 4 Department of Cognitive Science and Artificial Intelligence, Tilburg University, 5037 AB Tilburg, The Netherlands; 5 Donders Centre for Cognitive Neuroimaging, Radboud University, 6525 EN Nijmegen, The Netherlands; 6 Helen Wills Neuroscience Institute, University of California, Berkeley, CA 94720, USA; 7 Department of Psychology, University of California, Berkeley, CA 94704, USA

**Keywords:** random forest classifier, particle swarm optimization, eye tracking, spontaneous thought, mind wandering, multi-dimension experience sampling

## Abstract

One key aspect of the human experience is our ongoing stream of thoughts. These thoughts can be broadly categorized into various dimensions, which are associated with different impacts on mood, well-being, and productivity. While the past literature has often identified eye movements associated with a specific thought dimension (task-relatedness) during experimental tasks, few studies have determined if these various thought dimensions can be classified by oculomotor activity during naturalistic tasks. Employing thought sampling, eye tracking, and machine learning, we assessed the classification of nine thought dimensions (task-relatedness, freely moving, stickiness, goal-directedness, internal–external orientation, self-orientation, others orientation, visual modality, and auditory modality) across seven multi-day recordings of seven participants during self-selected computer tasks. Our analyses were based on a total of 1715 thought probes across 63 h of recordings. Automated binary-class classification of the thought dimensions was based on statistical features extracted from eye movement measures, including fixation and saccades. These features all served as input into a random forest (RF) classifier, which was then improved with particle swarm optimization (PSO)-based selection of the best subset of features for classifier performance. The mean Matthews correlation coefficient (MCC) values from the PSO-based RF classifier across the thought dimensions ranged from 0.25 to 0.54, indicating above-chance level performance in all nine thought dimensions across participants and improved performance compared to the RF classifier without feature selection. Our findings highlight the potential of machine learning approaches combined with eye movement measures for the real-time prediction of naturalistic ongoing thoughts, particularly in ecologically valid contexts.

## 1. Introduction

William James coined the term “stream of consciousness” [1] to refer to the flow of thoughts we experience during the waking moments of our lives. At any given moment, these thoughts can vary notably. For instance, thoughts may be in the form of images, sounds, or sensations; they may focus on the external world or inner experiences, ourselves or others, the past or future, and so on. The thoughts we experience can greatly influence our mood, well-being, and productivity. Certain thought types, such as off-task thoughts—which occur when attention shifts away from the task at hand—have been associated with negative affective consequences [2]. However, other dimensions of thought have been linked to positive affective consequences [3,4]. Indeed, the content of thoughts and the context in which they occur are associated with different outcomes [5,6,7,8,9], highlighting the need to distinguish between different dimensions of thought. Despite the functional significance of our ongoing thoughts, the detection of these various dimensions of thought remains largely unexplored.

The classification of thoughts requires a method that can capture the heterogeneity of thought content and form. To that end, multi-dimensional experience sampling (MDES) [10] has emerged as a useful approach for assessing ongoing thoughts. This approach involves using thought probes to sample one’s inner experience by asking a series of questions that capture multiple dimensions of thoughts. For example, these dimensions include task-relatedness, thought dynamics (freely moving or constrained), and modalities (visual or auditory). These thought probes are typically sent at pseudo-random intervals throughout a task in an experimental setting [11,12] or throughout the day in everyday life [13]. Such experience sampling methods have been used in conjunction with neuroimaging measures such as fMRI or EEG to determine neural markers of thought dimensions [9,11,14,15,16]. However, as these neuroimaging techniques can be difficult to implement in real-world settings, eye tracking has been established as a more scalable method for thought detection [17].

The well-established relationships between oculomotor behavior and various cognitive domains [18,19,20] suggest that eye movements offer unique insights into cognition. Similar to other cognitive domains, some thought dimensions have also been associated with patterns of eye movement, such as fixations and saccades. For example, off-task thoughts have been associated with fewer complex eye movements [21] compared to on-task thoughts. Similar to off-task thoughts, goal-directed thoughts in the context of idea generation have also been linked to reduced microsaccades [22]. Furthermore, internally-directed thoughts have been associated with fewer and longer fixations compared to externally-directed thoughts during a sentence generation task [23]. These prior studies have investigated correlates of individual thought dimensions in the context of an experimental task in the laboratory, which may limit generalizability beyond those highly controlled tasks. Global features from eye tracking data, which include oculomotor behaviors independent of specific tasks, show promise in detecting thoughts across diverse task contexts [24,25,26,27]. 

Past research on thought detection has generally assessed a limited number of thought dimensions during experimental tasks. Much of the literature exploring classification of thought has primarily focused on detecting the dimension of off-task thoughts during sustained-attention tasks or reading tasks (see [27] for a review), but such tasks are often not representative of the more complex and personally relevant tasks that individuals may encounter in their daily lives. Studies have shown that people are more likely to purposefully engage in certain thought types depending on task demands and context [11,16,25,28], suggesting that the types of ongoing thoughts elicited during a strictly controlled experimental task may be different from those that arise in our everyday lives. Therefore, it stands to reason that allowing participants to choose whichever task they wish to perform (thus mimicking their home office setting) would more likely elicit naturalistic ongoing thoughts. No studies to our knowledge have attempted to detect multiple dimensions of ongoing thoughts during naturalistic behavior using eye movements. 

The detection of thoughts using eye tracking has primarily been implemented with machine learning approaches [24,29,30,31,32,33] using classifiers such as logistic regression, Bayesian network, random forest (RF), and support vector machine. The RF classifier has previously shown high classification performance in detecting off-task thoughts using eye movements [30], particularly in comparison to support vector machine and logistic regression when using eye tracking measures alone [34]. Furthermore, an RF algorithm showed successful detection of internally directed thoughts compared to spontaneous and deliberate on-task thoughts [35], which suggests that the RF algorithm may be ideal for the classification of thought dimensions.

While machine learning algorithms can identify numerous features that can optimally detect naturalistic thoughts, additional feature selection techniques are necessary to identify the best subset of features that contribute most to classification performance. Among them, the particle swarm optimization (PSO) algorithm is considered to have a strong global search ability and has been widely used for solving feature selection problems. This algorithm’s effectiveness in identifying the best feature sets has been verified in various applications [36,37,38], with an eye tracking-based RF classifier optimized with PSO resulting in an accuracy of up to 96% for the classification of levels of attention to external tasks [39]. We therefore implemented the PSO algorithm for feature selection. Taken together, although past studies have combined machine learning with eye tracking data to detect a few thought dimensions (i.e., off-task thoughts and internally oriented thoughts) during experimental tasks, the gaze measures associated with most thought dimensions and their utility combined with machine learning to detect these thoughts are largely unknown. 

In this study, we developed and tested a PSO-based RF model to determine if eye movement data can be used as a robust and generalizable measure to classify thoughts across nine dimensions (task-relatedness, freely moving, goal-directedness, stickiness, internal–external orientation, self-orientation, others-orientation, visual modality, and auditory modality) during naturalistic tasks. Seven participants underwent seven experimental sessions during which they were occasionally prompted to report their ongoing thoughts during self-selected computer tasks while their eye tracking data were being recorded. Given the large number of thought dimensions assessed in this study, we prioritized acquiring more data per participant to obtain reliable estimates of eye tracking measures corresponding to thought dimensions, versus obtaining a small amount of data across a larger number of participants. As no experimental control was placed on the occurrence of a given thought dimension, it is possible that some dimensions of thoughts would not occur in a given experimental session. Therefore, our study acquired multiple sessions of data (approximately nine hours) from each participant to ensure that sufficient data exist for all thought dimensions, resulting in a total of 49 datasets. Classification of thoughts was based on statistical features extracted from eye movement measures (e.g., fixations and saccades), and model performance for the nine thought dimensions was assessed with the Matthews correlation coefficient (MCC). Several of the thought dimensions explored in this study have not been previously examined with eye tracking features or classified with gaze-based machine learning approaches. To our knowledge, this is the first study to implement gaze-based classification of a comprehensive set of thought dimensions using PSO in a naturalistic context. 

## 2. Materials and Methods

**Figure 1:**
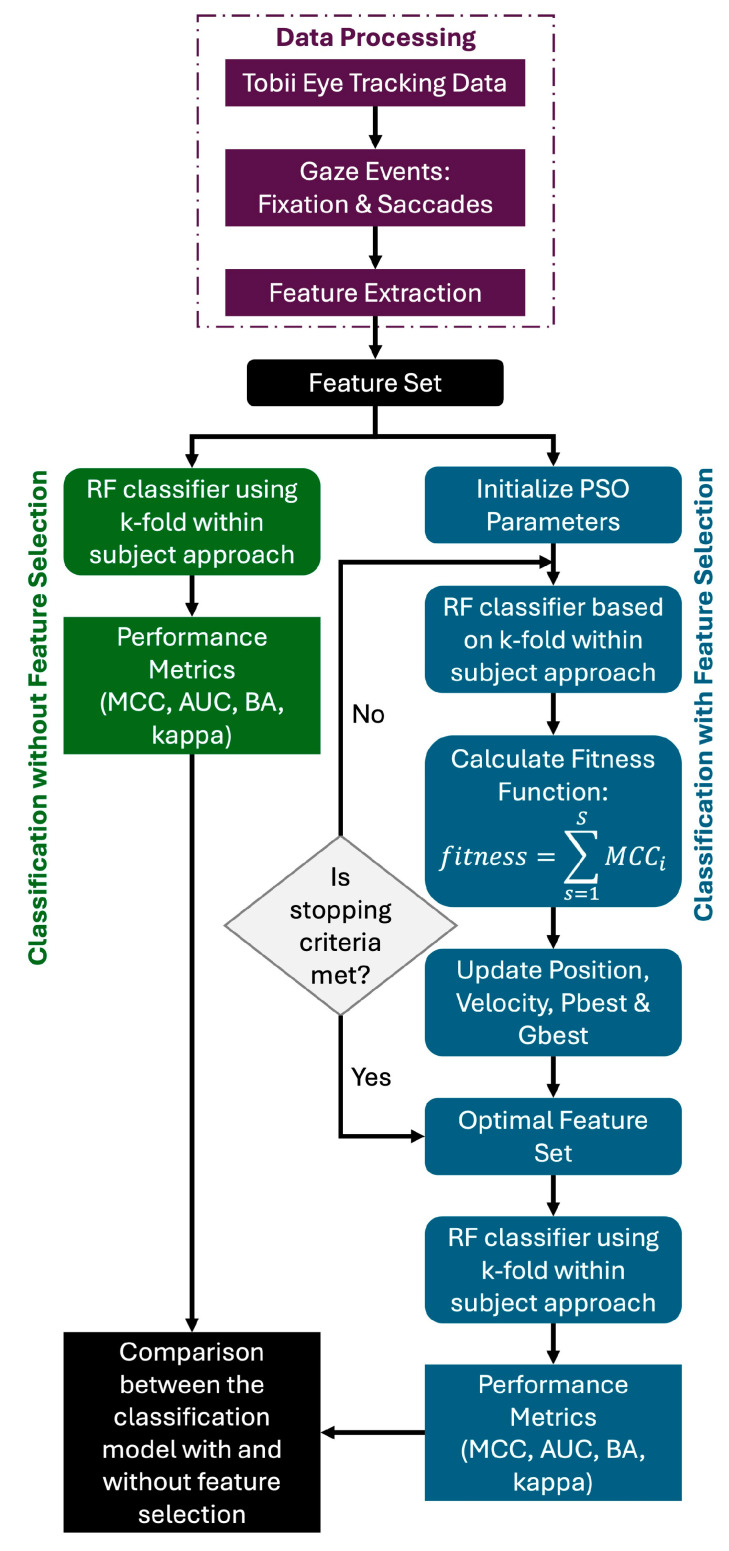
Comparison of MCC values of RF classifier with the optimal feature set and without any feature selection.

The overall block diagram is shown in Figure 1. Five modules such as data collection, pre-processing, feature extraction, feature selection, and classification were used in this process. They are briefly described in the following sections.

### 2.1. Participants

Seven participants (3 males and 4 females; age: mean = 24.57 years, STD = 3.66; years of education: M = 16.4, S.D. = 1.51) completed this study. They had no history of neurological disorders and had normal or corrected-to-normal vision. All participants provided written informed consent and were paid for their participation. This study was approved by the Conjoint Faculties Review Ethics Board (CFREB) at the University of Calgary.

### 2.2. Experimental Protocol

All participants completed seven recording sessions across several days, resulting in a total of 49 datasets. Before the start of this study, participants underwent a training session where they were familiarized with the terminology used in the thought probes as part of the experience sampling protocol (see Section 2.3). In particular, they were provided with definitions and examples of each thought dimension measured in the experiment and were given the opportunity to ask the experimenters clarifying questions about them during this training. Although several data streams were collected, only eye tracking data were reported in this study as the other data streams listed were reported elsewhere and are beyond the scope of this paper. Participants were seated at a desktop computer and were 60 cm away from an eye tracker attached to the bottom edge of the monitor screen. The height of the monitor was adjusted based on participant height. Eye tracking calibration was performed using the Tobii Pro Eye Tracker Manager (version 1.12.2). Participants were told to keep as still as possible during data recording. After calibration was completed, participants were instructed to complete whichever task they wished to perform on the computer for a period of approximately 80 min. During this time, we recorded their eye tracking data and occasionally prompted them to report their ongoing thoughts (as described in Section 2.3). There was a total of approximately nine hours of acquired data per participant. An experimenter checked the calibration of the eye tracker approximately 2–3 times per experimental session, and re-calibration was performed if the eye tracker was not correctly synced with eye movements. 

### 2.3. Thought Probes and Self-Assigned Task

Each participant was instructed to complete self-selected tasks on the computer during each of the experimental sessions. That is, participants were free to choose whichever task they wished to perform, in order to increase the ecological validity of our study to elicit ongoing thoughts that were more representative of daily life. With the increased prevalence of remote, computer-based work, we considered the self-selected “task” as a representation of everyday activity for participants. 

While participants were completing their self-selected tasks, multi-dimensional thought sampling (MDES) thought probes consisting of a list of questions were presented on the computer monitor. These occurred approximately every 120 s (with a range of 90–150 s to minimize expectation effects), with 35 thought probes presented during each experimental session. This amounted to a total of 245 thought probes across the seven sessions for each participant, resulting in a total of 1715 thought probes. The MDES thought probes were presented alongside a brief tone (1000 Hz, 200 msec) to alert participants of its occurrence. Each probe asked participants to answer several questions about their self-selected activity and ongoing thoughts during the time immediately preceding the tone. First, participants were asked to briefly report their task, defined as the self-selected activity they were supposed to be engaging in, as an open-ended question. They were then asked to describe the content and form of their thoughts across nine dimensions by responding to a 7-point Likert scale for the following questions: (1) To what extent were your thoughts on-task versus off-task?, (2) To what extent were your thoughts focused on your inner thoughts versus external stimuli?, (3) To what extent were your thoughts freely moving?, (4) To what extent were your thoughts goal-directed on a topic?, (5) To what extent did you have difficulty disengaging from your thoughts? (referred to as sticky thoughts hereafter), (6) To what extent were your thoughts about yourself?, (7) To what extent were your thoughts about another person/other people?, (8) To what extent were your thoughts in the visual modality (whether it be external or internal)?, and (9) To what extent were your thoughts in the auditory modality (whether it be external or internal)? Presentation of the thought probes was implemented through MATLAB 2021a [40].

Responses to each question were dichotomized for ease of quantifying responses and comparing the eye tracking measures (as described below). As an example, for the task-related question, a score of 1–3 would be considered on-task, while a score of 5–7 would be considered off-task, and a score of 4 was discarded and removed from subsequent analyses. We used their responses to each question to label the 12 s of data preceding the probe. That is, we extracted eye features (as described below) within the 12 s period prior to each thought probe and categorized them based on the thought probe response (e.g., on-task or off-task). We chose this 12 s window for several reasons. First, this aligned with previous studies using experience sampling to capture thoughts (e.g., [25,41]). Second, neuroimaging evidence has shown that markers of certain thought dimensions, such as off-task thoughts, were observed for 10–20 s prior to the thought probe response [42]. Third, this time window reflects a tradeoff between maximizing the amount of data included to obtain a reliable estimate of eye features while maintaining a reasonable validity of the participants’ report. 

The number of trials (corresponding to the number of thought probe responses) for each of the nine thought dimensions varied across participants. Some participants did not have any data points for a thought dimension, either due to a lack of reports in a given class of a thought dimension or a lack of usable data. For example, in some instances, participants did not look at the screen for a significant portion of time during the session; since features extracted in such a scenario would be incorrect, these data points were excluded. To ensure sufficient data in subsequent classification analyses, a threshold limit was set to include only those participants that had a minimum of 10 trials in each class for each thought dimension. The number of participants considered for classification analyses of each of the nine thought dimensions are reported in Table 1. The number of data points for each class of a given thought dimension for each participant is reported in Supplementary Table S1.

### 2.4. Eye Tracking Data Acquisition and Preprocessing

Eye tracking data were collected continuously using a Tobii Pro Fusion screen-based eye tracker (Tobii, Stockholm, Sweden) sampling at 250 Hz. The Tobii software outputted X and Y coordinates of participants’ gaze for each sample. These coordinates were represented in the form of proportions on the screen and were converted to pixel values for feature extraction. The screen size, pixel density, and participants’ distance from the screen remained consistent across all sessions, ensuring correct conversion to pixels.

We used the I-VT filter [43] to classify these data points into fixations (i.e., points in which gaze was maintained on the same location) and saccades (i.e., movement of the eyes between fixations). To classify eye movements, the I-VT filter associates a velocity value with each point by taking into account movement 10 ms before and after that point. Points were classified as fixations if the velocity of the eyes fell below the threshold of 30 degrees/second; if the velocity value was above 30 ms, that point was labelled as a saccade. In some instances, coordinate values were not available because participants blinked or looked away from the screen. Such instances were labelled as “unclassified”. Across all participants and sessions, 64% of points were classified as fixations, 26% as saccades, and 10% were unclassified. Successive fixation annotations that were at least 60 ms combined were marked as a single fixation. Successive saccade annotations that lasted at least 30 ms were combined into one [44]. These minimum values were set to match the default values used in the I-VT algorithm. Combining these points together allowed us to extract features from the gaze data. 

Finally, blinks were not included in our analysis for several reasons. First, there are no conventionally accepted minimum blink thresholds for blink extraction during continuous eye tracking [45]. Given that task type has an impact on blink durations [45], a fixed-threshold approach implemented across tasks may lead to incorrect blink extraction as participants performed a wide range of self-selected tasks in this study. Second, blink durations have not been robustly associated with task-unrelated thought [45] or other thought dimensions in the literature. Third, blinks are determined by missing eye tracking samples in our system; however, the loss of eye tracking samples may also be caused by participant head movements, or because of off-screen fixations. Considering that participants may have looked away from the screen for reasons not directly related to their self-assigned task (e.g., looking at the keyboard before typing), this measure of blinking may not reliably and selectively capture blinks. Taken together, blinks were considered less robust than fixations or saccades for the present study and thus were not included in subsequent analysis.

### 2.5. Eye Tracking Features

Given that participants were allowed to complete any tasks they wished, the external stimuli varied within and across sessions for each participant, rendering local features difficult to interpret. Therefore, we focused on global eye tracking features [24,31,46] that fell within the 12 s time window preceding each thought probe (as described above), with a specific focus on fixations and saccades. There were a total of 29 global eye tracking measures, including fixation duration, saccade duration, and saccade amplitude and velocity, from which the statistical measures of mean, median, minimum, maximum, range, and standard deviation were computed (Table 2). Other measures included fixation count, fixation root mean square deviation (RMSD), fixation–saccade ratio (which represents how often a participant engaged in fixations compared to saccades), saccade count, and horizontal saccades (Table 2). 

### 2.6. Machine Learning Model

To classify the different dimensions of thoughts using eye tracking measures, we used the random forest (RF) classifier [47,48] with a set of decision trees (DTs) and an ensemble learning approach. In an RF classifier, multiple DTs are used and the majority outcome of all the DTs serves as the outcome of the RF classifier. The process of combining various DTs to obtain the outcome in the RF classifier is based on bootstrap aggregation (or bagging) and random split selection. The RF method has been applied to eye tracking data [30,34] due to its ability to identify the main correlation features (i.e., features that highly correlate with each other) that provide higher classification performance with fast computation speed. 

Accordingly, this study used an RF classifier to classify thoughts across nine dimensions using eye tracking data. The number of learners was set as 100. The RF classifier was evaluated using the stratified k-fold within-subject (k-fold WIS) strategy with a 5-fold scheme, which ensured each fold has an equal proportion of data points for each class as the original dataset. During this process, each participant’s data points were randomly split into k (k = 5) smaller sets called folds [49]. In each round, one of the k folds acted as a test set and the remaining folds (k-1) acted as a training set. Given we set the k-value to 5, this reduces the number of data points included in each fold (compared to a smaller k-value). We therefore implemented several control analyses to address this issue, all of which are reported in the Supplementary Tables S2 and S3. We first computed the classification performance for each fold and then the mean performance across the 5 folds for each participant. Finally, we report the classification performance for each thought dimension as the mean classification performance averaged across all participants. 

As the number of data points between the two classes in each thought dimension was imbalanced, we implemented the synthetic minority over-sampling technique (SMOTE) to create a balanced dataset by oversampling the minority class in the training set. Balancing the training set mitigates the risk of overfitting the data and improves the generalizability of the classification model. The RF classifier was trained using the balanced training set to predict the binary class labels on the original (unbalanced) test set. To evaluate the performance of the RF classifier, we computed four performance metrics: Matthews correlation coefficient (MCC; range = −1 to 1, chance = 0), area under the curve (AUC; range = 0 to 1, chance = 0.5), balanced accuracy (BA; range = 0 to 1, chance = 0.5), and Cohen’s kappa coefficient (κ; range ≤ 0 (no agreement) to 1 (perfect)). Although we report four metrics to facilitate comparison with the existing literature, our main focus is on the MCC as this metric is not influenced by an imbalanced dataset [50] and has previously been used in the context of thought detection [51]. To obtain a more reliable estimate of the classification performance, we implemented 50 iterations and reported the mean, maximum, and minimum classification performance across all 50 iterations.

### 2.7. Particle Swarm Optimization

In addition to assessing the classification performance across all features, we used particle swarm optimization (PSO) to identify the subset of features that contributed the most to the detection of each thought dimension. As described above, PSO has a strong global search ability and is effective in finding the best feature sets and has been verified in various applications [36,37,38]. Compared to other feature selection approaches (such as sequential-based feature selection which adds or removes features sequentially and produces locally rather than globally optimal solutions), the PSO technique is a global search method that evaluates subsets of features by using randomization search tactics to explore an entire section of the solution space in order to find the optimal subset of features. Given its benefits, we implemented the PSO approach for feature selection. 

PSO consists of a swarm of particles, where each particle $\left(i\right)$ is considered as a solution for an optimization problem and a set of possible solutions is considered as a search area [52]. A position is allotted to each particle in an N-dimensional search area, and each particle moves in the search area in order to find the best solution (fitness value) under the guidance of two vectors, namely position vector $y_{i}=[y_{i1},y_{i2},\ldots ,y_{iN}]$ and velocity vector $v_{i}=[v_{i1},v_{i2},\ldots ,v_{iN}]$. As each particle moves in a search area to identify the best solution, it self-updates its position $\left(y_{i}\right)$ and velocity $\left(v_{i}\right)$ based on its own experience and exchanges information with its neighbours. During this process, each particle records its best solution as $P_{best,i}$ and the best solution among all the particles (swarm) is recorded as $G_{best}.$ While recording the $G_{best}$ and $P_{best}$ value, each particle keeps track of the positions and velocity associated with the best values. This recorded information along with the current position and velocity of each particle is used to update the coordinates of each particle in a search area to find the best solution. Mathematically, they are represented as follows:

(1) $$v_{in}^{t+1}=\left\{w\right.*\left.v_{in}^{t}\right\}+\left\{c_{1}\right.*r_{1}*\left.\left(P_{best,in}^{t}-y_{in}^{t}\right)\right\}+\left\{c_{2}\right.*r_{2}*\left.\left(G_{best,n}^{t}-y_{in}^{t}\right)\right\}$$

(2) $$y_{in}^{t+1}=y_{in}^{t}+v_{in}^{t+1}$$

where n represents the $n^{th}$ dimension in the search area, t represents the $t^{th}$ iteration, w represents the inertia weight, $c_{1}\;\mathrm{and}\;c_{2}$ represent the social learning factors called cognitive parameters and social parameters, and $r_{1}\;\mathrm{and}\;r_{2}$ represent the random values in the range of [0, 1]. 

The first part of Equation (1) is called momentum, which acts as the memory of the previous move and helps prevent the particle from drastically changing direction. The second and third parts of Equation (1) are, respectively, referred to as self-cognitive, which quantifies the particle’s performance relative to its own past performance, and social learning, which quantifies the particle’s performance relative to its neighbor’s performance. They both ensure each particle is kept close to the $P_{best}$ and $G_{best}$ positions. The number of iterations, number of particles, and social learning factors $\left(c_{1},\;\mathrm{and}\;c_{2}\right)$, were selected as 100, 20, and 1.4962 based on past work [53]. PSO was iterated until a stopping criterion was reached. Three stopping criteria were used in this study: (i) maximum iteration was reached, (ii) the best fitness value of MCC equal to 1 was reached, or (iii) the fitness value has not changed for a minimum of 10 iterations.

We applied the PSO algorithm to select the best subset of 10 features across all participants for each thought dimension to improve the discriminating power of the classifier. Among the four reported performance metrics, we chose MCC as the fitness function because it most optimally captures the classification performance as it is insensitive to imbalances in the dataset [51]. The mean MCC values across all participants considered for each thought dimension are used as a final fitness value. The fitness function used to select the most informative subset of features is as follows:

(3) $$fitness=\sum_{s=1}^{S}MCC_{s}$$

(4) $$where,\;MCC_{s}=\frac{\left(TP*TN\right)-\left(FP*FN\right)}{\sqrt{\left(TP+FP\right)\left(TP+FN\right)\left(TN+FP\right)\left(TN+FN\right)}}$$

where *TP*, *TN*, *FP*, and *FN*, refer to the number of true positives, true negatives, false positives, and false negatives. All analyses were implemented in MATLAB 2021b [54].

In addition to PSO, we also implemented another feature selection approach post-hoc as an exploratory analysis to determine whether it leads to similar classification performance. Specifically, we performed the out-of-bag predictor importance estimates to extract the 10 most important features for each thought dimension to feed into the RF classification model. The PSO approach resulted in superior classification performance compared to the out-of-bag predictor importance approach. We report these results in the Supplementary Table S4. 

## 3. Results

### 3.1. Participant Self-Selected Tasks

All participants reported performing similar self-selected activities on the computer, which included reading (32%), writing or editing (14%), watching videos (19%), browsing or surfing the internet (11%), other cognitively demanding tasks (e.g., coding, playing games such as Sudoku, or preparing PowerPoint presentations, 23%) and others (e.g., doing nothing; 1%).

### 3.2. Classification Performance Based on All Features

The 29 eye tracking features served as input to the RF classifier for the classification of thoughts in nine dimensions using the k-fold WIS strategy. The mean, maximum, and minimum values of the classification performance across 50 iterations are reported in Table 3. We consider these results as the baseline to which we compare the performance using the PSO-based feature selection approach. All nine thought dimensions attained an above-chance level classification performance, with the mean MCC values ranging from 0.13 to 0.42. The other-oriented thought dimension showed the highest performance with a mean MCC value of 0.42, followed by the internal–external thought dimension with a mean MCC value of 0.34.

### 3.3. Classification Performance Based on Optimal Subset of Features

**Figure 2:**
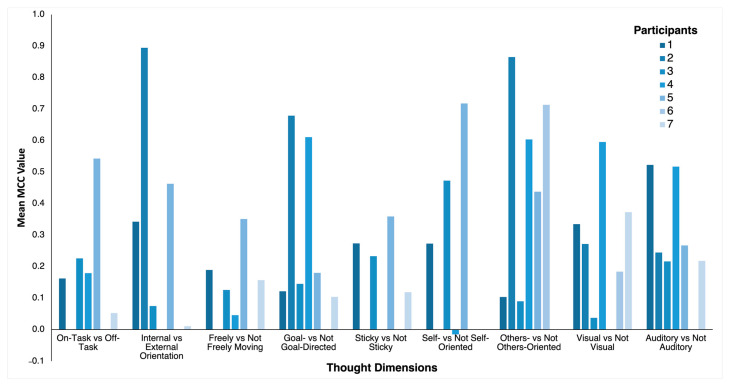
Mean MCC values for the nine thought dimensions for each participant. Only participants with sufficient data for classification for a given thought dimension were included in the analysis and shown here.

Next, we aimed to identify an optimal subset of 10 features using the PSO algorithm to further improve the prediction accuracy of the RF classifier. The 10 best features selected by the PSO algorithm for each thought dimension are reported in Table 4 and the mean classification performance averaged across participants based on this optimal feature set evaluated for 50 iterations is reported in Table 5. Similar to the above analyses, all nine thought dimensions attained an above-chance level classification performance, with the mean MCC values ranging from 0.17 to 0.47. The others-oriented thought dimension again achieved the highest mean MCC value of 0.47 and a maximum MCC value of 0.54. The internal–external thought dimension as well as the self-oriented thought dimension also performed well with both dimensions obtaining a mean MCC value of 0.36. In addition to group level classification performance, we also present individual level classification performance for each participant for each thought dimension in Figure 2. To compare the performance of the RF classifier to other common classification algorithms, we implemented the support vector machine and the k-nearest neighbor algorithms using the PSO-selected optimal feature set. These results are reported in Tables S5 and S6, respectively. The RF classifier outperformed both the support vector machine and the k-nearest neighbor classifiers. 

The mean values for each of the 10 PSO-selected features for each class of the thought dimensions are reported in Supplementary Table S7 offering insight into the specific eye movement correlates of each class of a given thought dimension. As these values were averaged across participants and sessions, they did not account for within and across individual differences and thus only serve to provide a crude estimate for interpretation purposes. They nonetheless shed light on the unique eye movement patterns associated with each thought dimension. For example, off-task thoughts were associated with increased fixation count and median fixation duration compared to on-task thoughts. Internally oriented thoughts were linked to decreased median saccade velocity and fewer horizontal saccades relative to externally oriented thoughts. Similarly, maximum fixation duration was reduced during freely moving thoughts, along with minimum saccade amplitude. Saccade count and median saccade velocity were reduced during goal-directed thoughts, whereas sticky thoughts were linked to a reduced fixation count and minimum saccade amplitude. Self-oriented thoughts were associated with a greater fixation range and mean saccade velocity; in contrast, fixation range and mean saccade velocity were reduced during others-oriented thoughts. Visual thoughts were linked to reduced minimum saccade amplitude and mean saccade velocity, while auditory thoughts correlated with higher median saccade amplitude and mean saccade velocity.

### 3.4. Comparison of Classification Performance 

**Figure 3:**
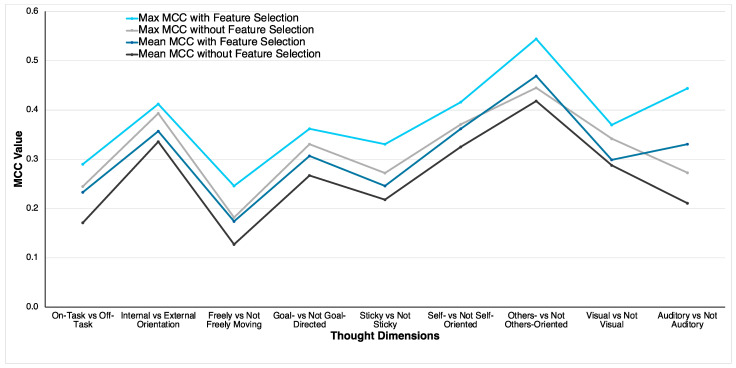
Comparison of MCC values of RF classifier with the optimal feature set and without any feature selection.

For all thought dimensions, the RF classifier attained better classification performance using the optimal feature sets. Specifically, the maximum MCC values attained by the optimal feature set were higher than the maximum MCC values attained by the RF classifier using all 29 features. The same pattern was observed for mean MCC values. A comparison of MCC values based on the two approaches based on the mean and maximum MCC values is illustrated in Figure 3. Similar figures illustrating the other performance metrics are reported in the Supplementary Material (Figures S1–S3). The mean MCC values attained by the optimal feature set for all the thought dimensions were 0.01 to 0.12 higher than the mean MCC values attained by the RF classifier without any feature selection. As an example, for the other-oriented thought dimension, the mean and maximum MCC values using the optimal feature set were 0.05 and 0.10 higher than the mean and maximum values attained using all the features. Using the optimal feature set, the largest improvement of 0.12 in the mean MCC value was observed in the auditory thought dimension. Together, these results indicate that the PSO-based feature selection algorithm showed a significant improvement in classification performance compared to classification without any feature selection. 

## 4. Discussion

In the current study, we tested the performance of a PSO-based RF classifier in detecting nine different thought dimensions (task-relatedness, internal–external orientation, freely moving, goal-directed, stickiness, self-orientation, others-orientation, visual modality, and auditory modality) using eye tracking measures acquired in a naturalistic task context across seven recording sessions. Our results showed above-chance level classification in all nine thought dimensions, with PSO-based feature selection further improving performance. Thus, our model demonstrates the utility of eye tracking approaches for classification of thoughts during naturalistic tasks by establishing a unique set of markers associated with each dimension.

Our study contributes to the literature in several ways. First, to enhance the ecological validity of our findings, participants in our study were free to choose whichever tasks they wished to perform, which enabled us to detect thought dimensions in a more naturalistic context. This also led to our focus on global eye tracking features that may be more generalizable across tasks, which contrasts with many previous studies focusing on task-dependent measures. Second, to our knowledge, we were the first to implement PSO to identify an optimal subset of interpretable features that led to enhanced detection performance in thought dimensions. This is consistent with previous research demonstrating superior classification performance based on a PSO approach compared to using all available features as input to the RF classifier [55]. Finally, we acquired data from seven participants across seven recording sessions, enabling us to gather a large amount of data for each thought dimension to obtain reliable estimates. This unique dataset contrasts with past studies with similar goals which primarily acquired single-session data [27]. Taken together, our PSO-based RF model achieved decent classification performance for all nine thought dimensions based on global eye tracking features in combination with statistical features, demonstrating the utility of eye tracking data for thought detection in more naturalistic contexts. We discuss specific findings, implications, and limitations of our findings below.

### 4.1. Task-Relatedness and Internal–External Orientation 

The optimal subset of features as chosen via PSO for the task-relatedness thought dimension included a combination of features linked to fixation duration (count, mean, median, minimum, and RMSD), saccade duration (minimum and standard deviation), and saccade velocity (maximum and range). In particular, we found that off-task thoughts were associated with a larger fixation count and longer median fixation duration compared to on-task thoughts. Of the thought dimensions explored in this study, the task-relatedness thought dimension has been the most commonly examined in the context of thought detection. The overall classification performance in our study is comparable to the performance reported in recent studies predicting on- vs. off-task thoughts using global and local eye tracking features during reading tasks (kappa = 0.15–0.45) [27]. Importantly, the PSO-selected features in this study were broadly consistent with the existing literature. For example, a previous study detected off-task thoughts during online lecture viewing using machine learning models based on global features from eye tracking, including statistical features calculated from fixation duration, saccade duration, saccade length, and velocity [31]. Similarly, another study found that fixation duration and saccade-based measures (specifically mean and maximum peak velocity, as well as maximum and mean amplitude) could be used to differentiate off-task from on-task states during video lectures [56]. The optimal set of features for detecting off-task thought in our study is consistent with these past studies. Other studies have noted more task-dependent relationships between off-task thoughts and associated eye tracking measures. For example, larger saccades were linked to off-task thoughts during auditory and sustained attention tasks whereas smaller saccade amplitudes and fewer fixations have been correlated with off-task thoughts during reading tasks [26]. The variable findings underscore the possibility of different global and local eye tracking features associated with off-task thoughts in a naturalistic context. In summary, our results corroborate past detection studies focusing on global eye tracking features. In light of the variety of tasks participants performed in this study, our results highlight the value of global eye tracking features as they are more generalizable across tasks.

The internal–external orientation thought dimension was best classified by a combination of features including fixation duration (median, maximum, range, and standard deviation), saccade duration (median and minimum), saccade amplitude (standard deviation), saccade velocity (median and maximum), and horizontal saccades. In particular, internally oriented thoughts were associated with reduced median saccade velocity and horizontal saccade count compared to externally oriented thoughts. These slower and fewer eye movements in internal orientation could reflect perceptual decoupling from external stimuli, as engagement in internal tasks has previously been linked with delayed saccades in a target–distractor task [57]. Thus far, no studies have attempted to detect this dimension of thought using eye tracking; however, a few experimental studies have examined the eye tracking correlates of internal–external orientation. For example, internally directed cognition has been connected to fewer and longer fixations [23], and fewer saccades [58], suggesting that this dimension is also associated with a unique eye tracking signature. Though the results of the present study are only partially in line with these prior findings, the global features found in this study may be more generalizable across task contexts. Importantly, the task-relatedness dimension is often thought to be associated with the internal–external orientation of thoughts, especially in a laboratory context in which on-task thoughts tend to be focused on an external laboratory task (e.g., reading, scene-viewing, sustained attention to response tasks). Though we found some common optimal features between these dimensions (median fixations, saccade duration minimum, and maximum saccade velocity), most features did not overlap. This highlights the importance of distinguishing between these two dimensions [59]. This is particularly important in ecologically valid contexts where a task may be internally oriented (e.g., doing mental math), whereas most experimental tasks tend to be externally oriented.

### 4.2. Freely Moving, Goal-Directedness, and Sticky Thoughts

According to the dynamic framework [60], freely moving thoughts move from one topic to another without an overarching goal or direction, and can be conceptualized as the opposite of goal-directed thoughts or sticky thoughts. This is because goal-directed thoughts are considered deliberately constrained via cognitive control and sticky thoughts are automatically constrained due to sensory or personally affective salience, whereas freely moving thoughts are associated with lower constraints.

Interestingly, there was some overlap in PSO-chosen features between all three dimensions (minimum fixation duration and standard deviation of saccade velocity), between freely moving thoughts and goal-directed thoughts (maximum fixation duration and minimum saccade duration), between freely moving thoughts and sticky thoughts (fixation count, standard deviation of fixation duration, and minimum saccade amplitude), and between goal-directedness and the sticky dimension (minimum saccade velocity). Despite these overlapping features between thought dimensions, they seem to be associated with different patterns for each dimension. For example, freely moving thought was associated with shorter fixation duration compared to non-freely moving thoughts, whereas this pattern was not observed in the goal-directedness dimension; this may suggest that the mind moving quickly between topics manifests in the frequency of eye movements. There were also features that were distinctively correlated with one dimension only, such as median saccade velocity and saccade count, which were linked with the goal-directedness dimension. In contrast, the sticky dimension was related to median saccade duration and median saccade amplitude. In summary, some features overlapped but not all features were shared across these dimensions, suggesting that, whereas different patterns on the same features can reflect all three dimensions, some features were uniquely associated with each of the three dynamic thought dimensions. 

### 4.3. Self- and Others-Oriented Thoughts

As with other dimensions explored in this study, the self and others orientation thought dimensions have not been previously explored with eye tracking in a naturalistic task context. Half of the features were similar between these two dimensions (minimum and range of fixation duration, fixation–saccade ratio, minimum saccade duration, and mean saccade velocity). Among these features, self-oriented and non-others-oriented thoughts both showed increased range of fixation duration and mean saccade velocity. However, they also have distinct features. For example, saccade range and saccade velocity range were associated only with the self-orientation dimension, whereas mean saccade amplitude and saccade count were only related to the others-orientation dimension. Since some thoughts may contain both self- and others-oriented thoughts, it is not surprising that some of these features overlap. Nonetheless, the features uniquely associated with each thought dimension also suggest self- and others-oriented thoughts are independent dimensions.

### 4.4. Visual and Auditory Modalities

There has been little research into the modality of thoughts as classified with eye tracking; however, prior works have noted that eye tracking may have potential in the context of studying mental imagery [61]. In our study, minimum fixation duration, minimum saccade duration, mean saccade velocity, and horizontal saccade count were common PSO-optimized features between visual and auditory thoughts, but the majority of features for each dimension did not overlap. For example, visual thoughts were linked to a reduced mean saccade velocity and minimum saccade amplitude, whereas auditory thoughts were associated with a higher median saccade amplitude and mean saccade velocity. Notably, a recent paper found eye movements to be the most common form of participant-reported detectable body movement associated with both visual and auditory mental imagery [62], which supports the notion that both thought modalities can be classified by unique eye movements.

### 4.5. Limitations, Future Directions and Implications

Several limitations need to be considered when interpreting these results. First, given that we aimed to collect a sufficient amount of data from each participant in the various thought dimensions by having multiple recording sessions, the sample size was limited to seven participants limiting the generalizability of our findings. This trade-off has been employed in other thought dimension studies to increase the signal-to-noise ratio in the data [63]. Future studies would need to recruit a larger number of participants to assess the generalizability of our findings as well as to examine potential individual differences in eye movement patterns associated with different thought dimensions across tasks. Second, as very few of the thought dimensions discussed have been previously explored with eye tracking measures, the interpretation of the individual features and any associated underlying cognitive processes remains speculative. Although examining the mean values of these eye tracking measures for each thought dimension suggests unique patterns associated with each thought dimension, these values were derived from obtaining the overall means across participants and sessions and did not consider within and across individual differences that were more precisely captured in the machine learning algorithms. A third point of consideration concerns potential systematic influences of tasks on the observed gaze patterns associated with a given thought dimension. Although we aimed to identify the optimal set of global eye tracking features of thought dimensions across tasks using PSO, studies have shown that these features are nonetheless influenced by task stimulus [26]. Future work should therefore consider systematically examining the features associated with each thought dimension that occurs during each type of task in more experimentally constrained settings. Fourth, unlike neuroimaging-based models such as those that employ EEG or fMRI, eye tracking-based thought classification does not inform the potential neural underpinnings of the various thought dimensions. Nonetheless, our study provides insight into how eye movements correspond with each thought dimension and demonstrates that classification performance with gaze-based measures are comparable to that with EEG and fMRI [49,53]. The portability, relative ease of implementation, and wide commercial availability of eye trackers and webcams also makes gaze-based classification a more feasible option for real-world implementation across task contexts. Finally, although above-chance classification was achieved with our model, it may be possible to improve performance by examining a different set of global eye tracking features or using different machine learning algorithms than the ones employed here. 

Despite these considerations, the present study is a proof-of-concept demonstrating the utility of eye tracking for thought detection across a comprehensive set of nine dimensions of ongoing thought. There are several important practical and clinical implications that inform future directions. To our knowledge, our results are the first to demonstrate that PSO can enhance performance of gaze-based thought detection, suggesting that PSO can be used to optimize features without reliance on the past literature to inform on relevant features. This is particularly important in the context of exploring thought dimensions that have not previously been detected with eye tracking, which is the case for several of the dimensions explored in this study. Future research may use these findings to develop algorithms that automatically detect thoughts in real-time. Given the challenge of employing neuroimaging markers of thoughts such as fMRI or EEG in daily life, our findings based on eye tracking measures may expedite the implementation of real-time thought detection, which has been successfully accomplished in off-task thought detection in an educational setting [33,64]. Moreover, such real-time algorithms could be used for detection of specific thought dimensions; specifically, thought identification could be used in conjunction with mindfulness or other cognitive control techniques to reduce negative thoughts or increase positive thoughts to improve well-being and productivity. Future investigations can also develop eye tracking-based biomarkers of disorders characterized by altered thoughts, such as attention-deficit hyperactivity disorder or depression [65]. 

## 5. Conclusions

In summary, this study demonstrated that gaze features can be used with a PSO-based random forest classifier to detect naturalistic thoughts with above chance performance in nine dimensions—task-relatedness, freely moving, stickiness, goal-directedness, internal–external orientation, self-orientation, others orientation, visual modality, and auditory modality. Each dimension was associated with a distinct set of optimized eye tracking features, which highlight the value of considering the heterogeneity of ongoing thoughts. This has implications for the implementation of thought tracking in real-time in daily life contexts and clinical settings.

## Figures and Tables

**Table 1:** Number of participants considered for classification of each thought dimension.

Thought Dimensions	Number of Participants Considered for Classification
On-Task vs. Off-Task	5
Internal vs. External	5
Freely Moving vs. Not Freely Moving	5
Goal-Directed vs. Not Goal-Directed	6
Sticky vs. Not Sticky	4
Self-Oriented vs. Not Self-Oriented	4
Others-Oriented vs. Not Others-Oriented	6
Visual vs. Not Visual	6
Auditory vs. Not Auditory	6

**Table 2:** Global eye tracking and statistical features used in the machine learning model.

Eye Tracking and Statistical Features	
fixation duration	count, mean, median, minimum, maximum, range, standard deviation, root mean squared deviation (RMSD)
saccade duration	count, mean, median, minimum, maximum, range, standard deviation
saccade amplitude	mean, median, minimum, maximum, range, standard deviation
saccade velocity	mean, median, minimum, maximum, range, standard deviation
fixation–saccade ratio	count
horizontal saccade	count

**Table 3:** Classification performance of the RF classifier using all 29 features.

Thought Dimensions	MCC			AUC			BA			kappa		
	Mean	Max	Min	Mean	Max	Min	Mean	Max	Min	Mean	Max	Min
On-Task vs. Off-Task	0.17	0.25	0.13	0.65	0.68	0.63	0.58	0.61	0.57	0.17	0.24	0.13
Internal vs. External Orientation	0.34	0.39	0.29	0.73	0.75	0.70	0.67	0.70	0.64	0.33	0.39	0.28
Freely vs. Not Freely Moving	0.13	0.18	0.06	0.60	0.63	0.56	0.56	0.59	0.53	0.12	0.18	0.06
Goal- vs. Not Goal-Directed	0.27	0.33	0.22	0.70	0.73	0.67	0.63	0.65	0.60	0.26	0.32	0.20
Sticky vs. Not Sticky	0.22	0.27	0.16	0.65	0.68	0.61	0.60	0.62	0.58	0.21	0.26	0.16
Self- vs. Not Self-Oriented	0.33	0.37	0.28	0.73	0.76	0.69	0.66	0.68	0.63	0.32	0.36	0.27
Others- vs. Not Others-Oriented	0.42	0.45	0.35	0.79	0.81	0.76	0.70	0.72	0.67	0.41	0.44	0.34
Visual vs. Not Visual	0.29	0.34	0.24	0.71	0.74	0.68	0.64	0.67	0.61	0.28	0.34	0.23
Auditory vs. Not Auditory	0.21	0.27	0.14	0.70	0.74	0.63	0.60	0.63	0.58	0.21	0.27	0.14

Note: MCC = Matthews Correlation Coefficient; AUC = Area Under the Curve; BA = Balanced Accuracy.

**Table 4:** The optimal subset of 10 features for each thought dimension using PSO.

Thought Dimensions	Optimal Features
On-Task vs. Off-Task	fixation (count, mean, median, min, RMSD); saccade (min, std), velocity (max, range); fixation–saccade ratio
Internal vs. External Orientation	fixation (median, max, range, std); saccade (median, min); amplitude (std); velocity (median, max); horizontal saccades
Freely vs. Not Freely Moving	fixation (count, mean, min, max, std); saccade (min, std); amplitude (min, std); velocity (min)
Goal- vs. Not Goal-Directed	fixation (min, max, RMSD); saccade (count, min, range); velocity (median, min, std); horizontal saccades
Sticky vs. Not Sticky	fixation (count, min, std); saccade (median); amplitude (median, min, range); velocity (min, max, std)
Self- vs. Not Self-Oriented	fixation (min, range); saccade (min, max, range, std); amplitude (median); velocity (mean, range); fixation–saccade ratio
Others- vs. Not Others-Oriented	fixation (min, range); saccade (count, median, min); amplitude (mean, std); velocity (mean, min); fixation–saccade ratio
Visual vs. Not Visual	fixation (min, max, std); saccade (min, max, std); amplitude (min, std); velocity (mean); horizontal saccades
Auditory vs. Not Auditory	fixation (count, mean, median, min); saccade (count, min); amplitude (median, max); velocity (mean); horizontal saccades

Note: min = minimum, max = maximum, std = standard deviation, RMSD = root mean square deviation; fixation and saccade refer to fixation and saccade duration. Amplitude and velocity refer to saccade amplitude and velocity.

**Table 5:** Classification performance of the RF classifier using the optimal feature set.

Thought Dimensions	MCC			AUC			BA			kappa		
	Mean	Max	Min	Mean	Max	Min	Mean	Max	Min	Mean	Max	Min
On-Task vs. Off-Task	0.23	0.29	0.17	0.68	0.71	0.64	0.61	0.64	0.58	0.23	0.28	0.17
Internal vs. External Orientation	0.36	0.41	0.31	0.72	0.75	0.70	0.68	0.71	0.65	0.35	0.40	0.31
Freely vs. Not Freely Moving	0.17	0.25	0.11	0.62	0.66	0.59	0.58	0.62	0.56	0.17	0.24	0.11
Goal- vs. Not Goal-Directed	0.31	0.36	0.26	0.71	0.74	0.68	0.65	0.68	0.62	0.30	0.36	0.25
Sticky vs. Not Sticky	0.25	0.33	0.18	0.65	0.68	0.61	0.62	0.66	0.58	0.24	0.32	0.17
Self- vs. Not Self-Oriented	0.36	0.42	0.31	0.73	0.77	0.69	0.68	0.71	0.65	0.36	0.41	0.30
Others- vs. Not Others-Oriented	0.47	0.54	0.41	0.80	0.82	0.77	0.73	0.77	0.70	0.46	0.54	0.41
Visual vs. Not Visual	0.30	0.37	0.24	0.73	0.75	0.69	0.65	0.69	0.62	0.29	0.36	0.24
Auditory vs. Not Auditory	0.33	0.44	0.23	0.75	0.79	0.72	0.66	0.72	0.61	0.32	0.43	0.23

Note: MCC = Matthews Correlation Coefficient; AUC = Area Under the Curve; BA = Balanced Accuracy.

## Data Availability

The data presented in this article are not readily available because these data are part of an ongoing study. Requests to access the data should be directed to the corresponding author.

## Supplementary Materials

The following supporting information can be downloaded at: https://www.mdpi.com/article/10.3390/bioengineering11080760/s1, Supplementary results: Post hoc analysis: exploring the number of data points per class and K-value on classification performance; Supplementary results: Post hoc analysis: exploring another feature selection approach; Table S1. Number of samples for each class of a thought dimension for each participant; Table S2. Classification performance of the random forest classifier using the optimal feature set for participants with a minimum of 16 data points in each class; Table S3: Classification performance of the random forest classifier using the optimal feature set with k = 2 to 5; Table S4. Features selected based on out-of-bag predictor importance approach; Table S5. Classification performance using the support vector machine algorithm with optimal features derived from the PSO approach; Table S6. Classification performance using the k-nearest neighbor algorithm with optimal features derived from the PSO approach; Table S7. Values of PSO-selected features for the thought dimensions, averaged across all participants and sessions; Figure S1. Comparison of area under the curve (AUC) values of the random forest classifier with the optimal feature set and without any feature selection; Figure S2. Comparison of balanced accuracy (BA) values of random forest classifier with the optimal feature set and without any feature selection; Figure S3. Comparison of Kappa values of random forest classifier with the optimal feature set and without any feature selection.
