# Supplementary material for: Gaze-Based Detection of Thoughts across Naturalistic Tasks Using a PSO-Optimized Random Forest Algorithm
Source: Bioengineering (Basel). 2024 Jul 27;11(8):760. doi: 10.3390/bioengineering11080760 (PMC11351278; doi:10.3390/bioengineering11080760)

Gaze-Based Detection of Thoughts Across Naturalistic Tasks Using a PSO-Optimized Random Forest Algorithm

Tarannum Rahnuma†, Sairamya Nanjappan Jothiraj†, Vishal Kuvar,
Myrthe Faber, Robert T. Knight, and Julia W. Y. Kam

Supplementary Materials

Supplementary Results

Post-Hoc Analysis: Exploring the Number of Data Points per Class and K-value on Classification Performance

Based on our k-fold approach, our dataset was divided into k=5 folds. As the minimum total number of trials was 10, there is a minimum of 2 data points in each class at each fold. Across iterations per participant, the number of data points of each class ranged from 2 to 43 in a test set and 8 to 179 in a train test. The number of data points for both classes for a given thought dimensions ranged from 31 to 47 in a test set, and 121 to 186 in the training set. Although we implemented SMOTE to achieve balanced classes, some of the data points in the original dataset for some classes of a thought dimension in some participants were comparatively lower. Therefore, we implemented two sets of control analyses to address this issue.

First, to determine whether the number of data points per class affected the classification performance, we only included datasets that had a minimum of 16 data points in each class (i.e., minimum of 3 or 4 data points in the test set of each class and minimum of 12 or 13 data points in the train set of each class). We then computed the mean classification performance based on these datasets across subjects for each thought dimension (as reported in Table S2). As observed in Table S2, the number of subjects included in the analysis is reduced for five out of the nine dimensions with this higher threshold of minimum number of data points. We therefore only report the results for these five dimensions, since the performance of the other four dimensions (internal-oriented, goal-oriented, sticky and other-oriented thought dimensions) would not have changed with the same number of participants/datapoints included in the analyses. In comparing this with the original classification performance based on datasets with less datapoints, there were no major differences in classification performance for the off-task and auditory thought dimensions. For the freely moving, self-oriented, and visual thought dimensions, there was a slight increase in the mean MCC value ranging from 0.024 to 0.126. This suggests that removing datasets with fewer data points increased the classification performance of some thought dimensions but not all.

Another approach to increase the number of data points in the test class is by varying the k value. Therefore, we implemented the analysis with k values varying from 2 to 4 to evaluate its impact on classification performance. A smaller k-value means a smaller number of folds, preventing datapoints to be divided into multiple sets per class, thereby resulting in more data points in the test set and less data points in the training sets. By varying k-values from 2 to 4 using the k-fold cross-validation approach, we can determine how including more (k=2) or less (k=4) data points in the test set impacts classification performance. Finally, we compared classification performance with k=2 to 4 to performance with k=5, which is our original approach based on past literature empirically demonstrating this k-value prevents high bias and high variance in test error rate estimates. These results are reported in Supplementary Table S3.

The classification performance with k=2, k=3, and k=4 were inferior to the classification performance with k=5 for all thought dimensions. In general, classification performance increased as k-value increased, with classification performance being worst at k=2 and best at k=5. Specifically, when the k-value increases, the number of training sets increased as well, which in turn increased classification performance in our study. Taken together, in line with previous literature [1], a k-fold cross-validation approach with k=5 led to optimal classification performance.

Post-Hoc Analysis: Exploring another Feature Selection Approach

We implemented the Particle Swarming Optimization (PSO) technique as our feature selection approach in the study. The optimal set of features were then entered into the Random Forest (RF) classification model. Past findings have reported that that implementing feature selection before training the RF classifier improved classification performance [2–4], as it removed the redundant information from the feature set. In particular, the authors have applied PSO approach for feature selection before training the RF classifier and proved that PSO based feature selection approach have increased the classification performance.

As an exploratory analysis, we implemented another feature selection approach to determine whether other approaches lead to similar classification performance. Specifically, we performed the out-of-bag (OOB) predictor importance estimates to extract the 10 most important features for each thought dimension to feed into the RF classification model. Specifically, this approach involves evaluating the feature importance through random permutation of the features in the OOB samples and measuring the amount of misclassification rate as compared to the OOB rate with all features intact. We entered all 29 features as input to determine which features were more important for detecting the target class through OOB approach and obtained the feature importance value for all the 29 features. The obtained features importance values were used to rank the features from best to the least important features. After ranking the features, the top 10 features were selected and implemented the same RF classification model as described in the main text on the 10 most important features for each thought dimensions. Classification performance results are reported in Table S4. Consistent with previous research [4], classification performance based on this approach resulted in less superior classification performance than the PSO approach. Classification performance using a PSO-based feature selection approach was better than the classification performance based on all 29 input features (as reported in the manuscript).

Post-Hoc Analysis: Comparing the Performance of RF classifier with Support Vector Machine and K-Nearest Neighbour

To compare the classification performance of RF with two other commonly used classification algorithms, we implemented the support vector machine (SVM) [1] and the k-nearest neighbor (KNN) [1] algorithms to classify nine different thought dimensions using the optimal feature set derived from the PSO approach. The SVM was implemented with a Gaussian radial basis function and the KNN was implemented with 5 nearest neighbors.

Results are reported in Supplementary Tables S5 and S6. It is clear that compared to SVM and KNN classifiers, the RF classifier reported in the main manuscript (Table 5) attained the best performance in our study. These results indicate that the RF classifier is the best machine learning model for detecting nine different thought dimensions using eye-tracking data.

References:

1. Dong, H.W.; Mills, C.; Knight, R.T.; Kam, J.W.Y. Detection of Mind Wandering Using EEG: Within and across Individuals. *PLOS ONE* **2021**, *16*, e0251490, doi:10.1371/journal.pone.0251490.
2. Chen, Y.-T.; Lee, H.-H.; Shih, C.-Y.; Chen, Z.-L.; Beh, W.-K.; Yeh, S.-L.; Wu, A.-Y. An Effective Entropy-Assisted Mind-Wandering Detection System Using EEG Signals of MM-SART Database. *IEEE J. Biomed. Health Inform.* **2022**, *26*, 3649–3660, doi:10.1109/JBHI.2022.3187346.
3. Zhou, J.; Wang, G.; Liu, J.; Wu, D.; Xu, W.; Wang, Z.; Ye, J.; Xia, M.; Hu, Y.; Tian, Y. Automatic Sleep Stage Classification With Single Channel EEG Signal Based on Two-Layer Stacked Ensemble Model. *IEEE Access* **2020**, *8*, 57283–57297, doi:10.1109/ACCESS.2020.2982434.
4. Kazerani, R. Improving Breast Cancer Diagnosis Accuracy by Particle Swarm Optimization Feature Selection. *Int. J. Comput. Intell. Syst.* **2024**, *17*, 44, doi:10.1007/s44196-024-00428-5.

Supplementary Tables

Table S1. Number of samples for each class of a thought dimension for each participant.

Thought Dimensions	P1	P2	P3	P4	P5	P6	P7
On-Task	149		165	209	113		153
Off-Task	47		36	14	55		49
Internal Orientation	110	87	176		40		62
External Orientation	73	106	27		129		117
Freely Moving	96		102	195	72		157
Not Freely Moving	102		49	13	90		52
Goal-Oriented	71	189	33	25	17		68
Not Goal-Oriented	126	19	162	199	161		134
Sticky	145		143		151		191
Not Sticky	43		49		45		26
Self-Oriented	138		62	210	135		
Not Self-Oriented	65		120	11	48		
Others-Oriented	141	185	138	43	112	149	
Not Others-Oriented	59	18	66	168	75	19	
Visual	94	155	109	78		13	84
Not Visual	100	29	74	136		139	100
Auditory	44	10	22	13	11		72
Not Auditory	153	188	185	212	168		113

Note: participants with insufficient datapoints for a given class of a thought dimension were excluded from the analysis, as indicated by the empty boxes.

Table S2. Classification performance of the Random Forest classifier using the optimal feature set for participants with a minimum of 16 data points in each class.

Thought Dimensions	Number of Participants (Participant Number)	Mean MCC	Mean MCC (original threshold in Table 1)
On-Task vs Off-Task	4 (P1, P3, P5, P7)	0.246	0.233
Freely vs Not Freely Moving	4 (P1, P3, P5, P7)	0.206	0.174
Self- vs Not Self-Oriented	3 (P1, P3, P5)	0.488	0.362
Visual vs Not Visual	5 (P1, P2, P3, P4, P7)	0.323	0.299
Auditory vs Not Auditory	3 (P1, 103, P7)	0.319	0.331

Table S3: Classification performance of the Random Forest classifier using the optimal feature set with k=2 to 5.

Thought Dimensions	Mean MCC with k=2	Mean MCC with k=3	Mean MCC with k=4	Mean MCC with k=5
On-Task vs Off-Task	0.195	0.201	0.219	0.233
Internal vs External Orientation	0.340	0.352	0.352	0.357
Freely vs Not Freely Moving	0.154	0.163	0.172	0.174
Goal- vs Not Goal-Directed	0.280	0.299	0.315	0.307
Sticky vs Not Sticky	0.205	0.223	0.234	0.246
Self- vs Not Self-Oriented	0.311	0.348	0.353	0.362
Others- vs Not Others-Oriented	0.421	0.455	0.468	0.469
Visual vs Not Visual	0.247	0.272	0.290	0.299
Auditory vs Not Auditory	0.245	0.292	0.317	0.331

Table S4. Features selected based on out-of-bag predictor importance approach.

Thought Dimensions	Features	Classification performance (MCC): feature selection via OOB predictor importance	Classification performance (MCC): feature selection via PSO
On-Task vs Off-Task	fixation (count, min, *max*, *RMSD*, *range*); saccade (min); amplitude (*min*, *std*); velocity (mean), *fixation-saccade ratio*	0.193	0.233
Internal vs External Orientation	fixation (*mean*, *min*, std, *RMSD*), saccade (min); amplitude (*median*); velocity (*mean*, median, *std*); *fixation-saccade ratio*	0.341	0.357
Freely vs Not Freely Moving	fixation (min, *range, RMSD*), saccade (min); amplitude (std); velocity (*mean*, *std*, *range*); *horizontal saccades; fixation-saccade ratio*	0.130	0.174
Goal- vs Not Goal-Directed	fixation (min, RMSD), saccade (count, min, *median*, *std*); amplitude (*min*); velocity (*mean*, min); *fixation-saccade ratio*	0.296	0.307
Sticky vs Not Sticky	fixation (min, max, *RMSD*, range), saccade (count, min); amplitude (*min*); velocity (mean, *median*); *horizontal saccades*	0.228	0.246
Self- vs Not Self-Oriented	fixation (*min*, *std*, *RMSD*), saccade (count, min); amplitude (*median*); velocity (mean, *median*, range); *horizontal saccades*	0.346	0.362
Others- vs Not Others-Oriented	fixation (count, min, *max*), saccade (*count*, *min*); amplitude (mean, min); velocity (*mean*, *median*); *horizontal saccades*	0.455	0.469
Visual vs Not Visual	fixation (*count*, *median*, *min*), saccade (*median*, min, max, *range*); amplitude (*median*, *range*); velocity (); *fixation-saccade ratio*	0.290	0.299
Auditory vs Not Auditory	fixation (median, min), saccade (*median*, min); amplitude (*median*, *std*); velocity (*mean*, *median*, *std*); fixation-saccade ratio	0.225	0.331

Note: min = minimum, max = maximum, std = standard deviation, RMSD = root mean square deviation; Fixation and saccade refer to fixation and saccade duration. Amplitude and velocity refer to saccade amplitude and velocity. Italics indicate features that were not common with the PSO selected features.

Table S5: Classification performance using the SVM algorithm with optimal features derived from the PSO approach.

Thought Dimensions	MCC			AUC			BA			kappa		
	Mean	Max	Min	Mean	Max	Min	Mean	Max	Min	Mean	Max	Min
On-Task vs Off-Task	0.12	0.18	0.05	0.61	0.65	0.54	0.55	0.57	0.52	0.11	0.16	0.05
Internal vs External Orientation	0.22	0.25	0.17	0.66	0.70	0.62	0.60	0.62	0.58	0.21	0.25	0.16
Freely vs Not Freely Moving	0.05	0.11	0.01	0.53	0.57	0.49	0.52	0.55	0.50	0.05	0.11	0.01
Goal- vs Not Goal-Directed	0.13	0.22	0.05	0.63	0.67	0.58	0.55	0.58	0.52	0.12	0.19	0.05
Sticky vs Not Sticky	0.15	0.21	0.11	0.67	0.71	0.64	0.57	0.59	0.55	0.15	0.20	0.10
Self- vs Not Self-Oriented	0.17	0.22	0.12	0.65	0.69	0.61	0.58	0.60	0.55	0.17	0.21	0.11
Others- vs Not Others-Oriented	0.08	0.14	0.00	0.55	0.59	0.49	0.53	0.56	0.50	0.07	0.14	0.01
Visual vs Not Visual	0.11	0.17	0.01	0.60	0.64	0.55	0.55	0.58	0.51	0.11	0.16	0.01
Auditory vs Not Auditory	0.10	0.15	0.05	0.59	0.62	0.54	0.55	0.57	0.52	0.10	0.15	0.05

Table S6: Classification performance using the K-NN algorithm with optimal features derived from the PSO approach.

Thought Dimensions	MCC			AUC			BA			kappa		
	Mean	Max	Min	Mean	Max	Min	Mean	Max	Min	Mean	Max	Min
On-Task vs Off-Task	0.10	0.16	0.05	0.58	0.62	0.53	0.57	0.61	0.53	0.09	0.14	0.05
Internal vs External Orientation	0.22	0.27	0.16	0.65	0.68	0.62	0.62	0.65	0.58	0.20	0.25	0.15
Freely vs Not Freely Moving	0.07	0.13	0.01	0.55	0.58	0.51	0.54	0.57	0.50	0.07	0.12	0.02
Goal- vs Not Goal-Directed	0.14	0.20	0.08	0.63	0.66	0.59	0.60	0.63	0.56	0.12	0.18	0.06
Sticky vs Not Sticky	0.21	0.26	0.15	0.66	0.69	0.62	0.63	0.66	0.59	0.19	0.24	0.14
Self- vs Not Self-Oriented	0.20	0.27	0.13	0.64	0.68	0.59	0.61	0.66	0.56	0.19	0.25	0.12
Others- vs Not Others-Oriented	0.06	0.12	-0.03	0.53	0.57	0.48	0.53	0.57	0.48	0.05	0.10	-0.02
Visual vs Not Visual	0.12	0.17	0.06	0.59	0.62	0.56	0.57	0.60	0.54	0.11	0.16	0.05
Auditory vs Not Auditory	0.10	0.15	0.05	0.59	0.63	0.54	0.56	0.59	0.52	0.09	0.14	0.04

Table S7. Values of PSO-selected features for the thought dimensions, averaged across all participants and sessions.

Thought Dimensions	Best Subset of Features via Particle Swarm Optimization (PSO)									
	fixationCount	fixationMean	fixationMedian	fixationMin	fixationRMSD	saccadeMin	saccadeStd	velocityMax	velocityRange	fixationSaccadeCount
On-Task	31.12	0.11	0.09	0.06	275.64	0.06	0.01	354.84	305.41	0.40
Off-Task	35.40	0.11	0.10	0.06	278.04	0.06	0.01	354.61	304.07	0.42
	fixationMedian	fixationMax	fixationRange	fixationStd	saccadeMedian	saccadeMin	amplitudeStd	velocityMedian	velocityMax	horizontalSaccades
Internal	0.09	0.24	0.17	0.04	0.03	0.02	4.58	105.15	329.88	1.57
External	0.09	0.28	0.22	0.05	0.03	0.02	4.80	122.19	349.28	1.71
	fixationCount	fixationMean	fixationMin	fixationMax	fixationStd	saccadeMin	saccadeStd	amplitudeMin	amplitudeStd	velocityMin
Freely Moving	32.23	0.11	0.06	0.27	0.05	0.02	0.01	0.63	5.04	49.24
Not Freely Moving	34.77	0.12	0.06	0.32	0.06	0.02	0.01	0.83	4.57	51.54
	fixationMin	fixationMax	fixationRMSD	saccadeCount	saccadeMin	saccadeRange	velocityMedian	velocityMin	velocityStd	horizontalSaccades
Goal-Directed	0.06	0.27	254.92	24.55	0.02	0.05	112.22	48.29	72.73	1.68
Not Goal-Directed	0.06	0.27	265.05	26.70	0.02	0.05	116.56	49.60	77.93	1.66
	fixationCount	fixationMin	fixationStd	saccadeMedian	amplitudeMedian	amplitudeMin	amplitudeRange	velocityMin	velocityMax	velocityStd
Sticky	30.66	0.06	0.05	0.03	4.45	0.63	19.12	48.69	364.07	82.09
Not Sticky	33.09	0.06	0.05	0.03	4.18	0.80	17.96	50.69	343.53	75.59
	fixationMin	fixationRange	saccadeMin	saccadeMax	saccadeRange	saccadeStd	amplitudeMedian	velocityMean	velocityRange	fixationSaccadeCount
Self-Oriented	0.06	0.23	0.02	0.07	0.05	0.01	3.46	130.10	263.64	0.43
Not-Self Oriented	0.06	0.21	0.02	0.07	0.05	0.01	3.26	122.05	268.97	0.41
	fixationMin	fixationRange	saccadeCount	saccadeMedian	saccadeMin	amplitudeMean	amplitudeStd	velocityMean	velocityMin	fixationSaccadeCount
Others-Oriented	0.06	0.17	26.00	0.03	0.02	4.38	4.00	117.12	47.35	0.45
Not Others	0.06	0.21	25.72	0.03	0.02	4.85	4.10	132.14	50.48	0.38
	fixationMin	fixationMax	fixationStd	saccadeMin	saccadeMax	saccadeStd	amplitudeMin	amplitudeStd	velocityMean	horizontalSaccades
Visual	0.06	0.23	0.04	0.02	0.07	0.01	0.51	4.47	127.00	1.48
Not Visual	0.06	0.25	0.04	0.02	0.07	0.01	0.57	4.47	132.38	1.43
	fixationCount	fixationMean	fixationMedian	fixationMin	saccadeCount	saccadeMin	amplitudeMedian	amplitudeMax	velocityMean	horizontalSaccades
Auditory	30.70	0.11	0.10	0.06	27.70	0.02	5.19	21.46	155.85	1.46
NotAuditory	30.31	0.11	0.09	0.06	25.40	0.02	3.57	17.02	130.33	1.75

Supplementary Figures

Figure S1. Comparison of Area Under the Curve (AUC) values of Random Forest classifier with the optimal feature set and without any feature selection.

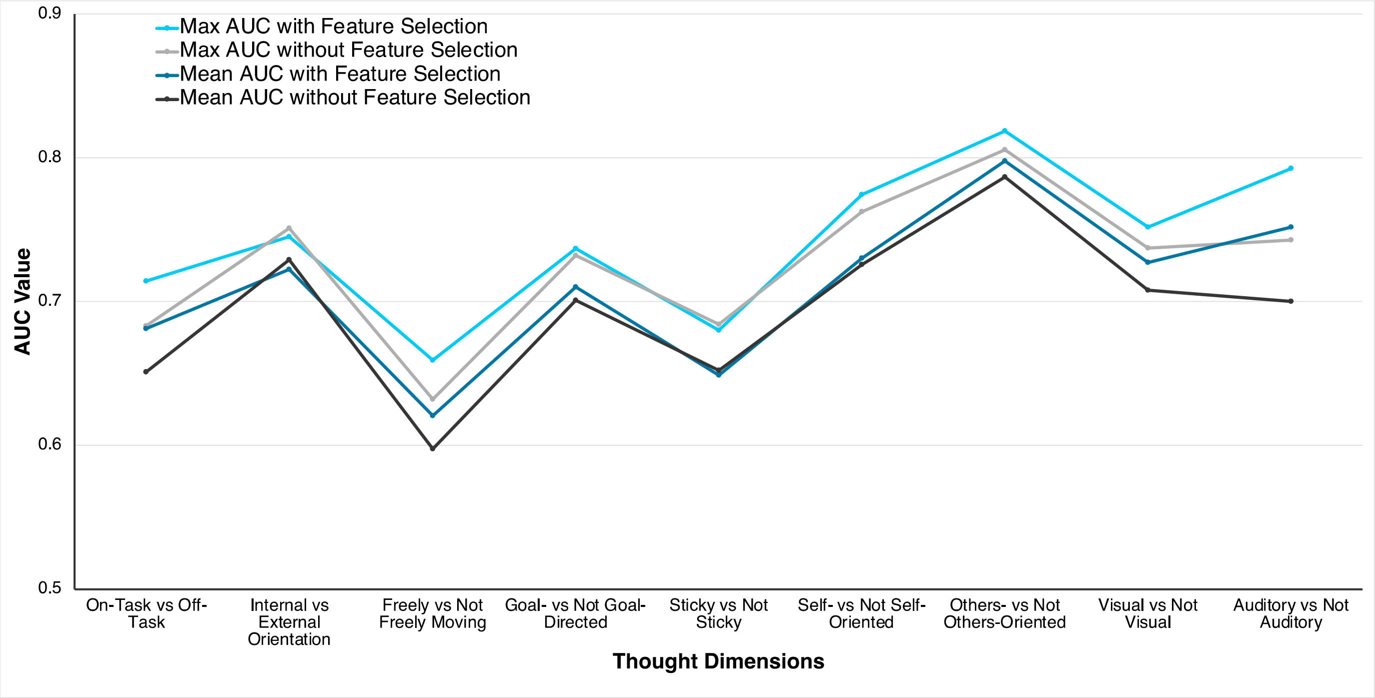

Figure S2. Comparison of Balanced Accuracy (BA) values of Random Forest classifier with the optimal feature set and without any feature selection.

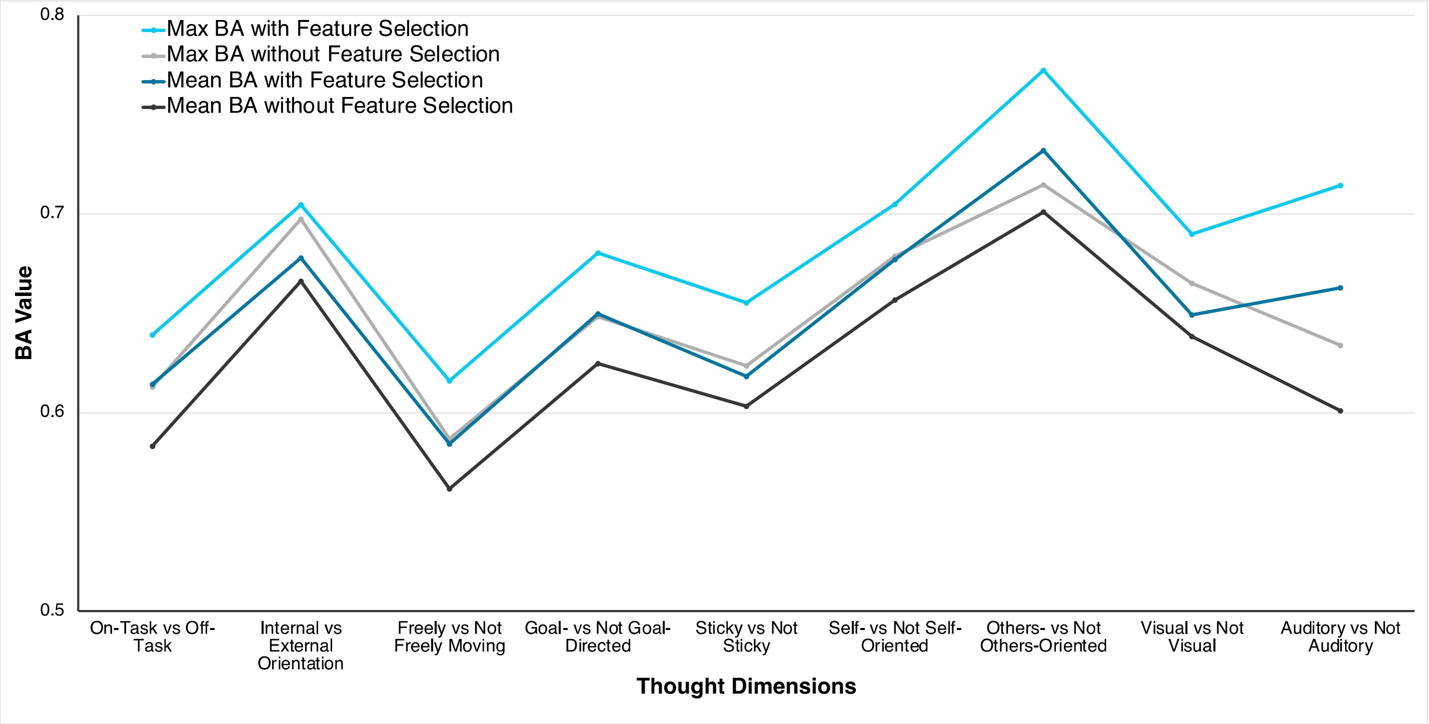

Figure S3. Comparison of Kappa values of Random Forest classifier with the optimal feature set and without any feature selection.

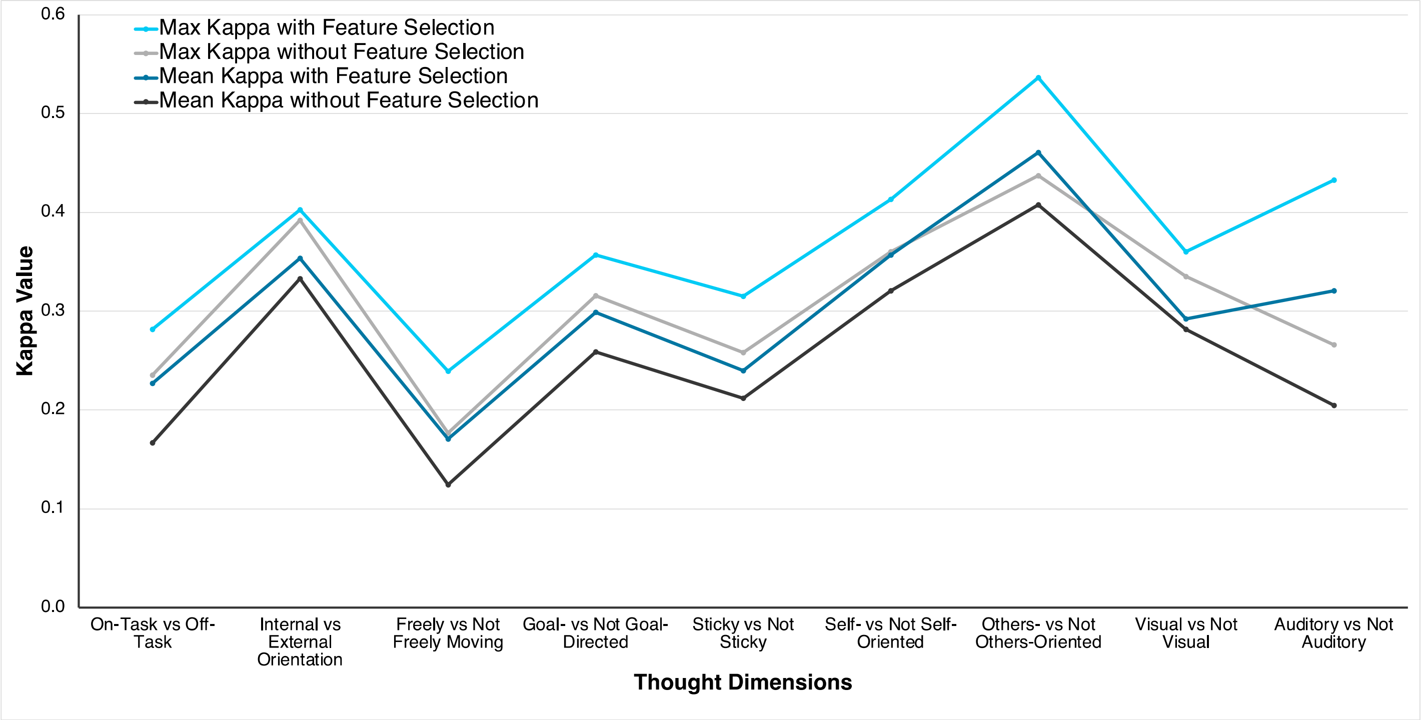

Supplement: Supplementary file 1 [file bioengineering-11-00760-s001.zip › bioengineering-3084783-supplementary.pdf]
